# Further Advances in Cancer Immunotherapy: Going Beyond Checkpoint Blockade

**DOI:** 10.3389/fimmu.2018.01082

**Published:** 2018-06-01

**Authors:** Robert W. Wilkinson, Andrew J. Leishman

**Affiliations:** Oncology Research, MedImmune Limited, Cambridge, United Kingdom

**Keywords:** immuno-oncology, toll-like receptors, oncology, Immunotherapy, tumour microenvironment

## Abstract

Significant advances have been made to identify effective therapies that either restore or generate *de novo* a patient’s immune response to cancer, so-called immunotherapy or immuno-oncology (IO) therapies. Some tumors overcome immune surveillance by promoting mechanisms to evade or suppress the immune system. This conference report highlights the clinical promise and current challenges of IO therapy, including the use of immune-checkpoint antagonist monoclonal antibodies. Furthermore, this report investigates advances in preclinical modeling of cancer immunobiology and how this is helping our understanding of which patients will receive clinical benefits from current immune-checkpoint treatment. Looking to the future, the report looks at emerging IO approaches, which aim to specifically target the tumor microenvironment. This includes the use of toll-like receptors (TLRs) agonists that link the activation of innate immune, cells to the priming of T cells and an adaptive memory anti-tumor immune response through to the reversal of local immunosuppression using adenosinergic and indoleamine 2,3-dioxygenase (IDO) inhibitors.

## Introduction

On 27th–30th June 2017, the 4th International Therapeutic Tolerance Workshop: First-in-Human Data was hosted by Newcastle University Institute of Cellular Medicine, UK. Session 2, Breaking Tolerance in Cancer, was Chaired by Andrew L. Mellor (Newcastle University). In this session, Robert W. Wilkinson (MedImmune Ltd., Cambridge, UK) gave a talk entitled “Immunological targets to combat Cancer,” a synopsis of his talk is described here.

The most clinically advanced immuno-oncology (IO) therapies are monoclonal antibodies (mAbs) that modulate the activity of T cells, by blocking inhibitory pathways that act as immunological checkpoints. The promising anti-tumor activity of mAbs targeting the immune-checkpoint proteins, such as cytotoxic T-lymphocyte antigen-4 (CTLA-4), programmed cell death protein 1 (PD-1), and the PD-1 ligand (PD-L1), led to regulatory approvals of these agents for the treatment of a variety of malignancies. The first of these drugs to be approved in 2011 was the anti-CTLA-4 antibody Ipilimumab (Yervoy^®^, Bristol-Myers Squibb) for the treatment of unresectable or metastatic melanoma (1). Subsequently, the anti-PD-1 mAbs, nivolumab (Opdivo^®^, Bristol-Myers Squibb) and pembrolizumab (Keytruda^®^, Merck & Co.) have gained regulatory approvals for the treatment of different cancers. More recently, clinical data with anti-PD-L1 antibody, durvalumab (Imfinzi^®^, MEDI4736), led to the approval for this drug in 2017 for the treatment of previously treated patients with locally advanced or metastatic urothelial carcinoma (2); further highlighting the potential of therapies that target immune evasion pathways.

## Current Understanding of Responses to IO Therapy

Immuno-oncology therapy has created a paradigm shift in the treatment of some advanced-stage cancers, where it is now the standard of care. However, while these agents can produce long-lasting responses in some cancer patients, the response rate as monotherapies tend to be low. A key goal now is to develop a deeper understanding of why some patients respond to IO therapies while others exhibit pre-existing immunological resistance, and may therefore be non-responsive to treatment, or become refractory (“acquire” resistance) to IO therapy with time. The immunological contexture of a patients’ tumor, the so-called “Immunoscore,” has been shown to be prognostic for outcome in several malignancies, including melanoma and colorectal cancer (3–5). These histological studies advance our understanding of how the immunological microenvironment of the tumor may impact patient outcome. Indeed, based on the wealth of data, there is now an argument for inclusion of immunoscore and immunoprofiling in standard disease staging, which is currently based on anatomical site, histopathology, and the characterization of defined genetic features, and by the incidence of local/distal metastasis. At a very basic level, tumors can be broadly described as “hot, cold, or immunosuppressive,” as determined by their profile of immune infiltrates. Tumors defined as “hot” are those with pre-existing tumor-infiltrating CD8^+^ cytotoxic T cells and natural killer (NK) cells. By contrast, “cold” tumors are poorly infiltrated by T cells, and “immunosuppressive” tumors, harbored high proportions of suppressive myeloid cells, such as myeloid-derived suppressor cells. Tumeh et al. recently reported a greater tumor infiltration with CD8^+^ cytotoxic T cells correlated with clinical responses to mAb’s targeting an immune checkpoint (6). Furthermore, Higgs et al. have showed high tumoural IFNγ mRNA and PD-L1 protein expression associates with response to durvalumab (anti-PD-L1 blocking mAb) monotherapy in NSCLC patients (7). Going forward, it is likely that a range of determinates and biomarkers will be incorporated to fully understand and predict responses to IO therapy, including the cancer patient’s somatic mutations and burden, tumor microenvironment (TME), and immune system characteristics.

## Recent Learning from Preclinical Mouse Models

To continue to advance the IO field, it will be important to use well-characterized and translationally relevant preclinical models. Currently, most IO therapies are tested in syngeneic transplanted mouse models of cancer, which means that the mice share a similar genetic background with the transplanted cells. The models are created by implanting a cancer cell line derived from a spontaneous, carcinogen-induced, or genetically engineered mouse tumor into an immunocompetent wild-type recipient. A survey of current literature points toward a lack of information about syngeneic tumor models, which potentially limits how well researchers can connect an IO therapy agent’s effects to its predicated impact in patients. MedImmune recently reported that they have built a large panel of murine syngeneic tumor models and profiled them in detail using readouts including copy number variation, exome mutations, transcriptomics, cytokine levels, and immune cell profiles within tumors and lymphoid organs (8). They went on to select six commonly used syngeneic mouse models and measured responses to anti-CTLA-4 or anti-PD-1 mAbs. While there was heterogeneity among the models they found, the strongest determinants of checkpoint inhibitor responses were the profiles of immune cells within the tumors, which broadly determined whether a model was “hot, cold, or immunosuppressive.” The “hot” cancer models (including, CT26 colorectal and RENCA kidney cancer models) were most responsive to anti-CTLA-4 and anti-PD-1 mAbs, a result that aligns with clinical evidence. Having a deeper understanding of the phenotype of preclinical models and how they relate to their human counterparts is helping to select optimal models to test preclinical hypotheses. For instance, the “cold” and “immunosuppressive” models will be valuable resources for groups developing IO therapies to overcome immunosuppression in the TME, such as cancer vaccines.

## Going Beyond Immune-Checkpoint Blockade

**Figure 1:**
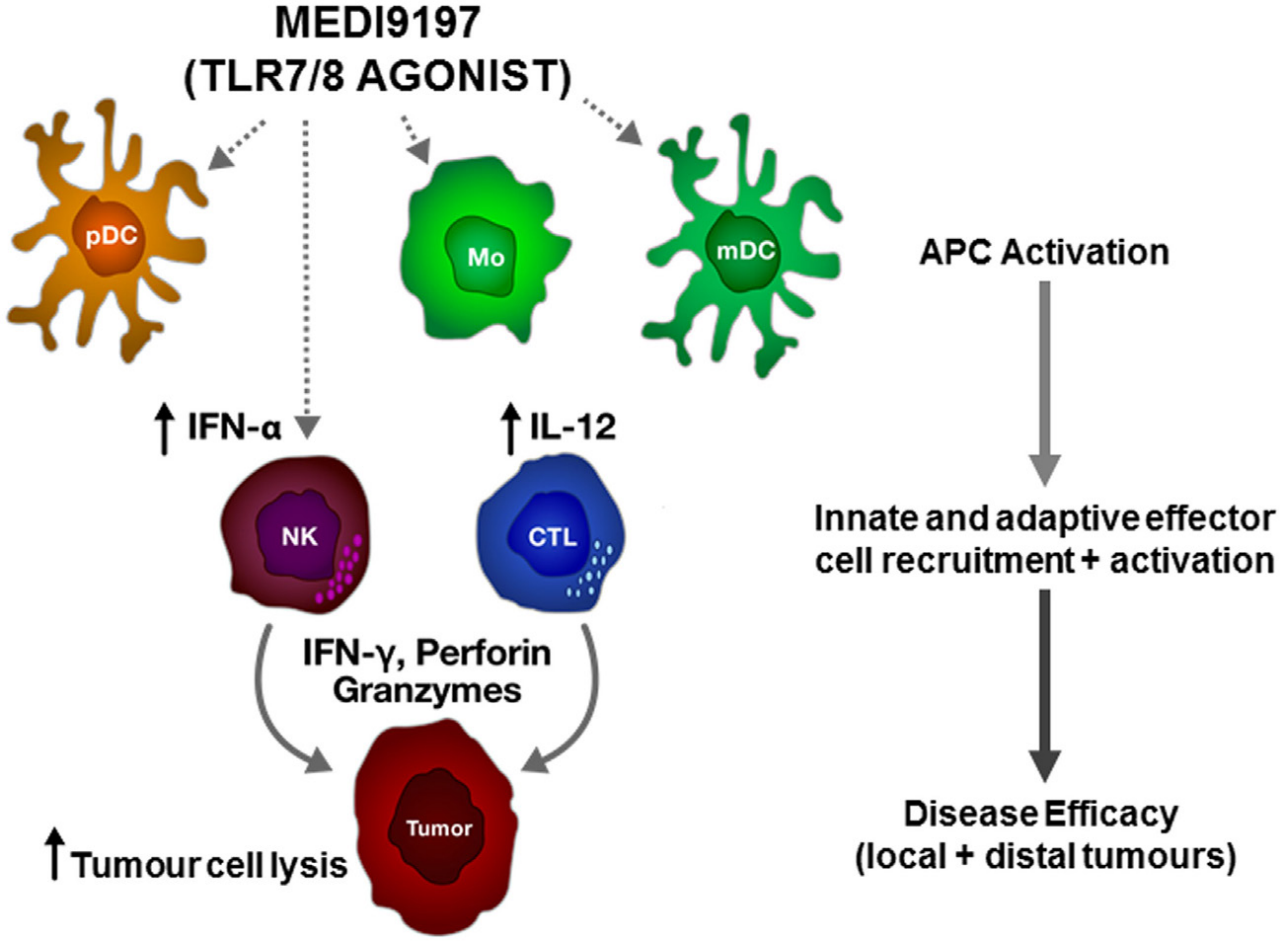
Proposed mechanism of action of MEDI9197 following intratumoural administration. MEDI9197 activates toll-like receptor (TLR) 7 and 8 expressing cells, such as plasmacytoid dendritic cells (pDC), myeloid dendritic cells (mDC), and monocytes (Mo), which release type I interferons and pro-inflammatory cytokines, such as interleukin-12 (IL-12); leading to recruitment and activation of effector cells, including natural killer (NK) cells and cytotoxic T lymphocytes (CTL) to the tumor. The activated effector cells release interferon gamma (IFN-γ), perforin, and granzymes to kill the tumor cells.

In addition to immune-checkpoint mAbs, there are a number of novel IO therapeutic approaches being developed to treat cancer patients. Toll-like receptors (TLRs) are expressed on a broad range of myeloid cells and function to recognize conserved pathogen-associated molecular patterns. Signaling through TLRs leads to the activation of antigen-presenting cells and to expression of inflammatory cytokines. MEDI9197 is a potent TLR7 and TLR8 agonist and induces pro-inflammatory cytokines through activation of myeloid and lymphoid cells (Figure 1). Preclinical mouse studies indicate that intratumoural injection of MEDI9197 induces a local inflammatory response, characterized by upregulation of genes associated with the activation of innate and adaptive immunity in the tumor (9). Importantly, in mouse syngeneic models that respond poorly to mAbs targeting either PD-L1 or CTLA-4, combination with MEDI9197 significantly improved anti-tumor activity when compared to either monotherapy alone. MEDI9197 is currently being evaluated in human clinical trials as a monotherapy in subjects with solid tumors and in combination with durvalumab and/or palliative radiation in subjects with solid tumors (NCT02556463). Preliminary data in patients indicate that MEDI9197 induces pharmacodynamic effects consistent with its expected mechanism of action (10).

Other IO therapeutic approaches aimed at reversing immunosuppression in the TME include blocking generation of the immune suppressive factor adenosine and its associated pathway. CD73 is an ectoenzyme that generates adenosine *via* adenosine monophosphate (AMP) hydrolysis. MEDI9447 is an example of an anti-CD73 mAb capable of relieving AMP-mediated lymphocyte suppression *in vitro* and inhibition of mouse syngeneic tumor growth *in vivo* (11) and is currently being evaluated in the clinic (NCT02503774). Interestingly, preclinical studies targeting the adenosinergic pathway by co-inhibition of CD73 and A2A adenosine receptor signaling improves anti-tumor immune responses, including limiting metastasis (12). Another metabolic pathway implicated in immunosuppression is indoleamine 2,3-dioxygenase (IDO), which promotes tolerance by catabolizing the amino acid tryptophan and other indole compounds (13). Indeed, preclinical studies targeting the IDO pathway have gained much attention for their clinical potential, as an immune-checkpoint inhibitor, in overcoming tumor-induced immunosuppression (14).

## Summary

Significant advances have taken place in our understanding of the interplay between cancer and the immune system, including therapeutic intervention using IO therapies. Our understanding of which patients will benefit from IO therapy continues to evolve, alongside our understanding of how best to modulate the anti-cancer immune response through combinations with other IO therapies and/or standard of care treatments.

## Author Note

4th International Therapeutic Tolerance Workshop, 27th–30th June 2017 at Newcastle University, UK; Session 2—Breaking Tolerance in Cancer. Chaired by Prof. Andrew Mellor, Newcastle University.

## Author Contributions

All authors listed have made a substantial, direct, and intellectual contribution to the work and approved it for publication.

## Conflict of Interest Statement

RW is an employee and shareholder of AstraZeneca/MedImmune. AL was a contractor at AstraZeneca/MedImmune at the time of the original submission of this article and is a shareholder of AstraZeneca/MedImmune. The reviewer RP and handling Editor declared their shared affiliation.
